# Fungal diversity and metabolomic profiles in GM and isogenic non-GM maize cultivars from Brazil

**DOI:** 10.1007/s12550-020-00414-8

**Published:** 2020-10-12

**Authors:** A. M. Gasperini, E. Garcia-Cela, M. Sulyok, A. Medina, N. Magan

**Affiliations:** 1 Applied Mycology Group, Environment and AgriFood Theme, Cranfield University, Cranfield, Bedfordshire, MK43 0AL UK; 2 Biological and Environmental Sciences, School of Life and Medical Sciences, University of Hertfordshire, Hatfield, AL10 9AB UK; 3 Institute of Bioanalytics and Agro-Metabolomics, Department of Agrobiotechnology (IFA-Tulln), University of Natural Resources and Life Sciences, Konrad Lorenzstr. 20, A-3430 Tulln, Vienna Austria

**Keywords:** Fungal diversity, Mycotoxigenic fungi, Metabolomics, Toxic secondary metabolites, Selective media, Maize cultivars, GM, Non-GM

## Abstract

There is little knowledge of the microbial diversity, mycotoxins and associated secondary metabolites in GM maize and isogenic non-GM cultivars (cvs). This study has quantified the microbial populations and dominant fungal genera in 6 cvs of each type representative of herbicide, pesticide or stacked resistance to both. The predominant mycotoxins and targeted metabolomics profiles were also compared between the two sets of cvs. This showed that the overall fungal populations were 8.8 CFUs g^−1^ maize. The dominant genera, isolated from maize samples, whether surface-sterilised or not, in all maize cvs were *Fusarium*, followed by *Penicillium*, *Aspergillus* and occasionally *Cladosporium* and *Alternaria.* The analysis of the targeted metabolomics showed that approx. 29 different metabolites were detected. These were dominated by fumonisins and minor *Penicillium* spp. metabolites (questiomycin A and rugulovasine A). Interestingly, the range and number of mycotoxins present in the GM cvs were significantly lower than in the non-GM maize samples. This suggests that while the fungal diversity of the two types of maize appeared to be very similar, the major contaminant mycotoxins and range of toxic secondary metabolites were much lower in the GM cvs.

## Introduction

In many parts of the world, maize production has become dominated by GM cultivars (cvs) which have replaced conventional ones, especially for improving herbicide and pesticide resistance. However, fungal pathogens of maize are responsible for significant economic losses in terms of yield and nutritional quality, especially during the critical silking period up to harvest. The major foliar pathogens include rusts, leaf spots, anthracnose, mildews and ergot. In addition, infection of ripening cobs by *Aspergillus*, *Penicillium* and *Fusarium* species can cause significant quality losses as well as contaminating them with toxic secondary metabolites (mycotoxins; Battilani et al. 2013). This results in *Aspergillus* ear or kernel rot (caused by *A. flavus*) and *Fusarium* ear rot (caused by *F. verticillioides*, *F. proliferatum*, and *F. subglutinans*), *Gibberella* ear rot (caused by *F. graminearum*) (Munkvold 2014). The *Aspergillus* section *Flavi* group contaminates the maize grain with aflatoxins, *Aspergillus* section *Circumdati* species and *Penicillium verrucosum* with ochratoxins, and the *Fusarium* species with either fumonisins or type B trichothecenes. There are legislative limits for some if not all these mycotoxins worldwide, with the most stringent regulations in the EU.

In Brazil, maize represents an important economic and social product in both family farming and agribusiness (Vidal et al. 2015). The country is the second largest biotech crop producer, after the USA, and is emerging as a global leader in this sector. Soybean production is the highest, followed by maize. Brazil has been a leader in the development of different biotechnology-modified traits, including 39 for maize (ISAAA 2017). Maize is cultivated in the summer and winter months in Brazil, with many differences between the management during the cropping season. All three categories of GM-type maize, insect resistance (IR), herbicide tolerance (HT), and the stacked IR/HT, are cultivated in both summer and winter maize.

Thus, in Brazil, GM maize cvs have become common and have largely superseded the equivalent conventional ones. Indeed, the tropical and sub-tropical climatic regions in Brazil are favourable for mycotoxigenic fungal colonisation of maize both pre- and post-harvest. Contamination of maize with aflatoxins and fumonisins has thus been frequently reported in Brazil (Salay and Zerlotti Mercadante 2002; Kawashima and Valente Soares 2006; Moreno et al. 2009; Caldas and Oliveira 2012; Baquião et al. 2012)

The adoption of GM crops continues to increase on a global scale, and the effects on mycotoxin contamination has been to a large extent ignored (Wu 2006). It has been reported that when plants were infested with Southwestern corn borers, a GM (*Bt*11) hybrid had > 75% reduction in aflatoxins when compared with its non-Bt counterpart (Windham et al. 1999). In Brazil, despite the large GM maize production, few surveys have investigated the similarity and differences in fungal diversity of the harvested maize grain. In addition, a relative comparison of mycotoxins and related toxic secondary metabolites of GM maize cvs with herbicide tolerance, insect resistance or both herbicide tolerance + insect resistance, and their original non-GM isogenic cvs has not been previously examined.

The objectives of this study were to examine harvested maize grain of 6 GM and their related non-GM isogenic cvs to compare (a) moisture content when harvested and stored, (b) fungal populations and the fungal diversity and (c) mycotoxins and related secondary metabolite profiles.

## Materials and methods

### Maize samples

The maize samples were harvested in Brazil in two seasons (2015 and 2016) in two different states (location 1 - Paraná, location 2 - Mato Grosso). Location 1 is a humid sub-tropical zone with average temperature ≥ 25 °C in the harvest season. Location 2 has a tropical climate with an average temperature ≥ 28 °C in the harvest season. A total of six pairs of maize (conventional and its GM isogenic cv; *n* = 12) of approximately 1 kg each cv were obtained. They were obtained by randomly taking smaller samples from different harvested maize plots of each cv and mixed to obtain the representative sample of each one. The samples were placed in sealed bags after harvesting and drying and sent by a courier to the Applied Mycology Group, Cranfield University, England, and stored at 4 °C until analysis. The details of the different Brazilian maize cvs examined are shown in Table 1.

**Table 1:** Description of the conventional (non-GM) and respective genetically modified (GM) isogenic cultivars of maize grain used in this study

Conventional cultivars (non-GM)	Isogenic GM cultivar	Traits tolerance present in the GM cvs
AS 1555 CON	AS 1555 PRO®	Pesticide-tolerant
BM-709 CON	BM-709 PRO_2_®	HT - Glyphosate R - Lepidopteran
CD-384 CON	CD-384 PW®	HT - Glyphosate HT - Glufosinate ammonium IR - Lepidopteran
M20-A78 CON	M20-A78 PW®	HT - Glyphosate HT - Glufosinate ammonium IR - Lepidopteran
P30F53 CON	P30F53 H®	HT - Glufosinate ammonium Antibiotic resistance IR - Lepidopteran
P2530 CON	P2530 Hx®	IR - Lepidopteran

*IR*, insect resistance; *HT*, herbicide tolerance

### Measurement of moisture content of the maize samples

Three 10-g sub-samples of each cv were weighed and placed in glass vials. These were dried in an oven at 110 °C for 24 h. Thereafter, they were placed in a desiccator jar containing silica gel and left to cool and the final dry weight obtained. The percentage moisture content (%MC) was then calculated on a wet weight basis.

### Fungal isolation from the maize samples

#### Enumeration of fungal populations

The enumeration of fungi was done using the serial dilution technique based on the method of Mohale et al. (2013). Three sub-samples of each maize cv (10 g) were soaked for 3 h in a sterile Stomacher bag containing 90 mL of sterile distilled water supplemented with 0.05% (w/v) technical agar (Oxoid, Basingstoake, UK) and 0.025% (w/v) Tween 80. The bags were homogenised for 5 min at high speed (300 rpm ± 5%) in a Stomacher blender (Lab-Blender 400; Seward Medical, UK). The sample bags were transferred in beakers to the sterile flow bench and allowed to settle for 5 min. Then, initially, using a 5-mL sterile tip, 1 mL was transferred to the 10^−2^ dilution bottle containing 9 mL of sterile water + 0.01% tween 80. Subsequently sterile 1-mL pipette tips were used to for serial dilution to 10^−3^ to 10^−5^. Between each serial dilution the 25 mL Universal bottles were shaken vigorously for 60 s. In the reverse order, 10^−5^ to 10^−1^, aliquots of 100 μL from each dilution were taken using a sterile 200-μL pipette tip placed centrally on the surface of the triplicate Petri dishes for each dilution. This was spread with a surface-sterilised L-shaped glass rod on the Malt Extract Agar (MEA; Thermo Fisher Scientific Oxoid Ltd., Basingstoke, Hampshire, UK) and Dichloran-Glycerol 18% agar (DG18; Thermo Fisher Scientific Oxoid Ltd., Basingstoke, Hampshire, UK) media.

The preparation of each growth media was done according to the manufacturers’ instructions using deionised water (15 Ωm). The media was autoclaved at 121 °C for 15 min at 103 kPA, and chloramphenicol (Fisher Bioreagents, Pasley, UK) was used as an anti-bacterial agent prior to autoclaving the media. The molten media were poured into 9-cm Petri dishes (approx. 17.5 mL per dish).

The Petri dishes (9 cm∅) were incubated for 7 days at 25 °C. The colonies growing in a range of 10 to 100 colonies were counted in three plates per dilution, and their numbers expressed as Log_10_ colony forming units (CFUs) per gramme dry weight of maize (Log_10_CFUs g dry weight^−1^). To obtain the actual fungal load, the calculated CFUs were adjusted based on the actual dry weight of the maize kernels after drying and reported as CFUs g^−1^ dry weight (Mohale et al. 2013).

#### Frequency of isolation of fungi

From each sample, 100 maize kernels were sub-sampled from each cv. Fifty (50) kernels from the sub-sample were first surface-disinfected (+SD) with sodium hypochlorite 0.4% v/v (NaOCl) for 2 min, left to dry and then plated, five maize kernels equidistant from each other on each Petri dish. The remaining kernels were plated without surface disinfection (−SD) in the same way. The kernels were directly plated (25 kernels per medium; five kernels per Petri dish) on DG18 and MEA media. The Petri dishes were incubated at 25 °C for 7–10 days, then inspected visually for fungal growth.

The fungal occurrence, i.e., number of maize grains from which *Aspergillus* sections *Flavi*, *Nigri*, and *Circumdati*, *Penicillium* and *Fusarium* and other fungi grew, was noted. To obtain the isolation frequency (%), all fungal colonies growing from directly plated kernels on MEA and DG18 were recorded.

### Identification of mycotoxins and other targeted metabolomic profiles of the GM and non-GM maize cultivars using LC-MS/MS

A multi-targeted metabolomics approach was used to identify the mycotoxins present in the maize samples. For these studies, the analysis was performed in duplicate for each cv because of the limited amount of maize available. The milled sub-samples (5 g) of maize were extracted using 20 mL extraction solvent (acetonitrile: water: acetic 79:20:1 (v/v/v) followed by a 1 + 1 dilution using acetonitrile: water: acetic 20:79:1 (v/v/v). Five microliters of the diluted extract was directly injected into the sampling port for LC-MS/MS in the equipment for analysis (Sulyok et al. 2020). A QTrap 5500 LC-MS/MS System (Applied Biosystems, Foster City, CA) equipped with a Turbo Ion Spray electrospray ionisation (ESI) source and a 1290 Series HPLC System (Agilent, Waldbronn, Germany). Chromatographic separation was performed at 25 °C on a Gemini® C_18_-column, 150 × 4.6 mm i.d., 5-μm particle size, equipped with a C_18_ 4 × 3 mm i.d. security guard cartridge (all from Phenomenex, Torrance, CA, USA). The chromatographic method and the chromatographic and mass spectrometric parameters were previously described by Malachová et al. (2014) and Sulyok et al. (2020). ESI-MS/MS was performed in the time-scheduled multiple reaction monitoring (MRM) mode both in positive and negative polarities in two separate chromatographic runs per sample by scanning two fragmentation reactions per analyte.

Quantification of the secondary fungal metabolites and mycotoxins was performed via external calibration using serial dilutions of a multi-analyte standard stock solution. The method covered all the mycotoxins addressed by regulatory limits as well as a range of other secondary metabolites. The reference standards for mycotoxins and other fungal metabolites are detailed by Sulyok et al. (2020). Results were corrected for recoveries obtained during method validation. The accuracy of the method has been verified on a continuous basis by regular participation in proficiency testing schemes (Sulyok et al. 2020). This approach has been previously applied to targeted metabolomics in cereal samples (Garcia-Cela et al. 2018).

### Statistical analysis and data sets

Data sets were subjected to Shapiro-Wilk tests to determine normality and Levene’s test to assess the equality of variance. The percentage moisture content (%MC) satisfied the two assumptions after transformation to cube root. Afterwards, one-way analysis of variance (ANOVA) was performed. The colony forming units (CFUs), frequency of fungal isolation and secondary metabolites data violated the two assumptions of ANOVA even after transformations, and consequently non-parametric tests (Wilcoxon/Kruskal-Wallis; *p* = 0.05) were used for analyses (Chan and Walmsley 1997). Where there was significance after the Kruskal-Wallis test, median comparisons for each pair of the different cvs were made using the Wilcoxon - Each Pair test (*p* = 0.05), while significance in ANOVA was done by comparisons of the means using Tukey HSD (*p* = 0.05). The statistical package JMP®14 (SAS Institute Inc., 2018, Cary NC, USA) was used to perform the analyses.

All the primary data sets from this manuscript are held by Cranfield University and are openly accessible via the corresponding author.

## Results

### Moisture content of the GM and non-GM cultivars

**Fig. 1:**
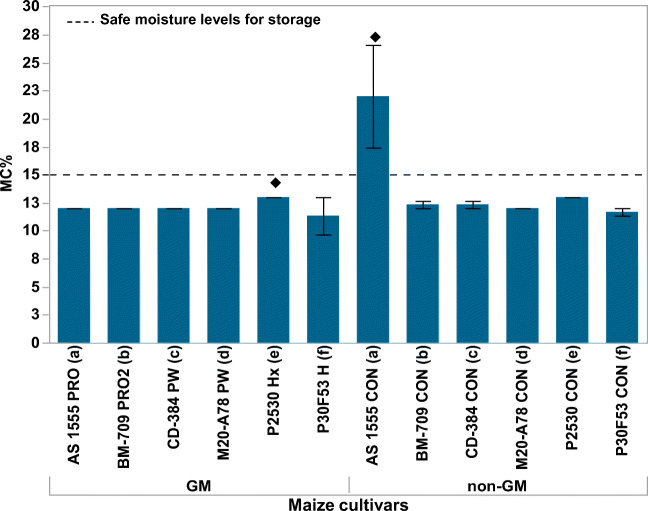
Values of moisture content (MC, %) of the GM and non-GM isogenic samples of maize cultivars. Bars represent the SE. The () shows those cultivars which were significantly different when compared with the other cultivars using the Tukey-Kramer HSD (*p* < 0.05)

The moisture content (MC, % wet weight) was between 11 and 22% (Fig. 1). The recommended conditions for safe storage of maize are MC ≤ 15% (= ≤ 0.70 water activity). Most of the samples were within the safety levels for storage without any potential for mould spoilage initiation. Significant differences at Tukey-Kramer HSD (*p* < 0.05) were found for two of the 12 cvs.

### Enumeration of the fungal populations isolated from the GM and non-GM maize samples

The GM and non-GM maize samples examined in this study had relatively high levels of total culturable fungal populations. The overall total fungal populations isolated from all cvs were < 8.8 log_10_ CFUs g^−1^ (Table 2). There was no significant difference in the populations (CFUs) between the samples at the 5% significance level on either MEA or DG18 media.

**Table 2:** Enumeration (log_10_CFU g^−1^ dry sample) of total fungal populations isolated from the genetically modified (GM) and non-GM cultivars

Type	Cultivar	MEA	DG18
GM	^1^AS 1555 PRO®	7.19 ± 0.03	7.17 ± 0.06
	^2^BM-709 PRO_2_®	6.87 ± 0.05	6.90 ± 0.05
	^3^CD 384 PW®	6.72 ± 0.15	5.97 ± 0.01
	^4^M20-A79 PW®	7.05 ± 0.08	6.91 ± 0.15
	^5^P2530 Hx®	8.14 ± 0.03	8.04 ± 0.15
	^6^P30F53 H®	8.10 ± 0.03	7.93 ± 0.09
non-GM	^1^AS 1555 CON	8.26 ± 0.02	8.27 ± 0.02
	^2^BM-709 CON	7.65 ± 0.04	6.98 ± 0.39
	^3^CD 384 CON	7.19 ± 0.03	7.73 ± 0.04
	^4^M20-A79 CON	7.28 ± 0.06	8.23 ± 0.03
	^5^P2530 CON	8.33 ± 0.04	8.79 ± 0.04
	^6^P30F53 CON	7.77 ± 0.02	7.73 ± 0.10

Values correspond to average ± SD (*n* = 3). Six isogenic lines were examined. No significant differences were found at 5% significance (Wilcoxon/Kruskal-Wallis test)

*MEA*, malt extract agar; *DG18*, Dichloran 18% Glycerol media

### Frequency of isolation of dominant fungi

**Fig. 2:**
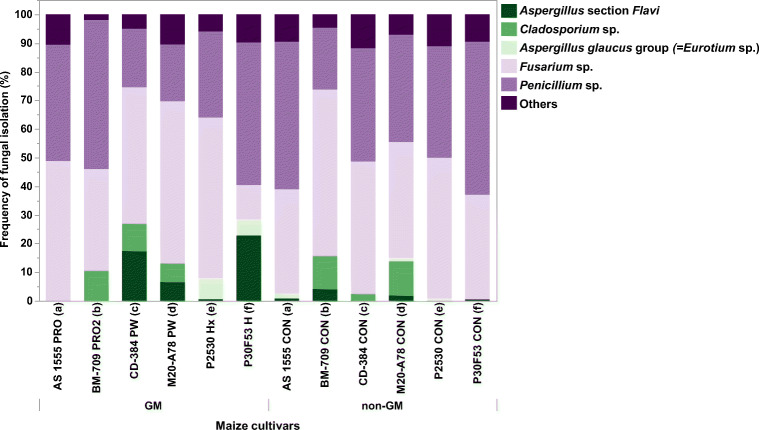
Relative frequency of isolation (%) of different predominant fungal genera isolated from the GM and isogenic non-GM maize cultivars. “Others” include species from the *Rhyzopus*, *Epicocum*, *Cladosporium*, *Mucor*, *Alternaria*, *Wallemia* and *Trhichoderma* genera. This is the combined data from both DG18 (Dichloran 18% Glycerol) and MEA (malt extract agar) media and includes the data for non-surface-sterilised and surface-sterilised maize kernels for each cultivar

**Fig. 3:**
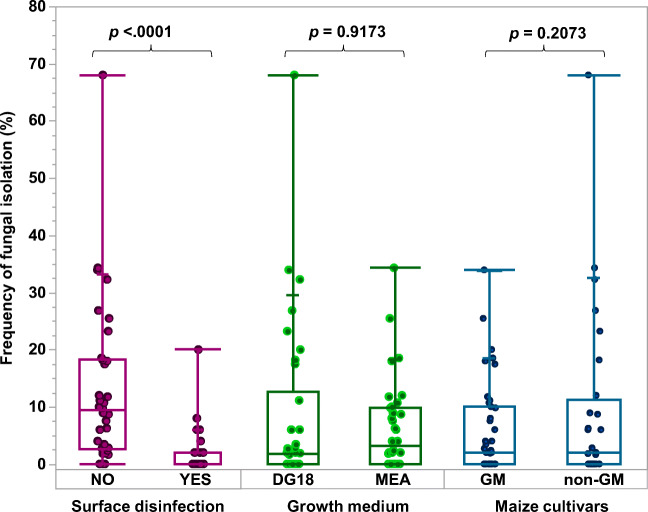
Overall frequency of fungal isolation (%) comparing non-surface-disinfected maize kernels (NO) with surface disinfection (YES); growth medium DG18 (Dichloran 18% Glycerol) vs MEA (malt extract agar); and GM vs non-GM cultivars. *p* < 0.05 denotes significant difference between the levels of isolation using the Wilcoxon - Each Pair test.

Figure 2 shows the relative frequency of isolation of different dominant fungal genera from the different GM and non-GM cvs when plated without or with surface disinfection on both media. There was a higher frequency of isolation from the non-disinfected plated maize kernels than from those that were surface-disinfected (Fig. 3). The surface disinfection allowed the isolation of internal fungal colonisers from the maize kernels when directly plated on the two media. The mycological analysis showed that *Fusarium*, *Penicillium* and *Aspergillus glaucus* group (= *Eurotium* species*)* were the principal contaminating genera of the maize kernels from both GM and non-GM cv samples. *Aspergillus* section *Flavi* strains were isolated from eight of the 12 cv samples. There was no significant difference (*p* = 0.05) in the frequency of isolation of the different fungi on the two-culture media (MEA × DG18) used or between non-GM and GM cvs (see Fig. 3).

### Mycotoxins and other secondary metabolites found in GM and non-GM cultivars of maize

**Fig. 4:**
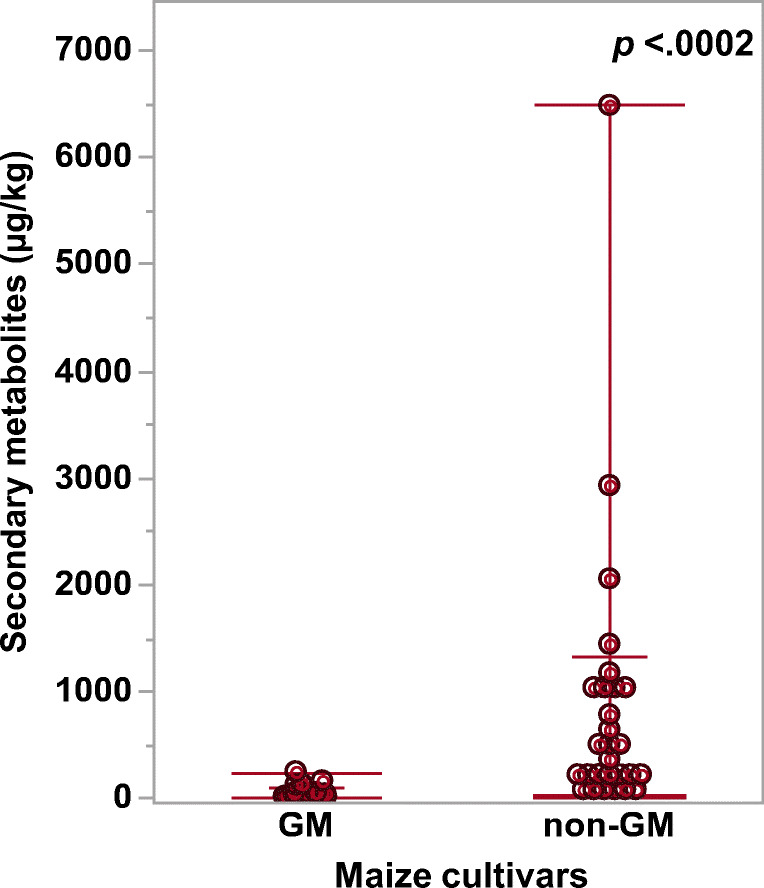
Overall distribution of all mycotoxin and related secondary metabolites identified from the GM and non-GM maize cultivars. *p* < 0.05 indicates significant difference between the levels using the Wilcoxon - Each Pair test

The LC-MS/MS analysis of the six GM and their isogenic non-GM maize cvs showed a higher presence of mycotoxins related to *Fusarium* and *Penicillium* spp., while toxins produced by *A. flavus* were largely absent. A total of 29 secondary metabolites were detected in the samples (Table 3). Mycotoxins of relevance in terms of food safety such as deoxynivalenol, zearalenone, trichothecens, ochratoxin A, citrinin or sterigmatocystin were not detected in the samples used in this study. An overall comparison of metabolites detected in the samples indicated that although there was no significant difference in the frequency of isolation (percentage; Fig. 3), the presence of mycotoxins was higher in the non-GM cvs (Fig. 4). Two regulated toxins (fumonisin B_1_ and B_2_; EC (2006)) were detected in higher amounts in the non-GM cvs. Comparing each cv individually, it was possible to identify marked differences between the GM and their isogenic non-GM line (*p* < 0.05) (see Table 3). For example, the non-GM cv (CD-384 CON) had the highest levels of fumonisin B_1_ (6480.5 μg.kg^−1^), while for its GM isogenic line (CD-384 PW®), the same toxin was not detected. A similar trend was found when comparing the non-GM line (P30F53 CON) where fumonisin B_1_ was > 5000 μg.kg^−1^, while in the GM line (P30F53 H), the toxin concentration was about 40× lower (148 μg.kg^−1^).

**Table 3:** Mycotoxins and related secondary metabolites (μg.kg^−1^) found in the GM and non-GM maize cultivars using LC-MS/MS.

Group	Compounds	AS 1555 CON*	AS 1555 PRO••	BM-709 CON*	BM-709 PRO_2_••	CD-384 CON*	CD-384 PW••	M20-A78 CON*	M20-A78 PW••	P2530 CON*	P2530 Hx••	P30F3 CON*	P30F53 H••
A	^♦^Fumonisin B_1_	110	-	1060	125	6480	-	168	-	-	24.7	5550	148
	^♦^Fumonisin B_2_	26.4	-	464	46.8	2050	-	53.3	16.9	16.3	-	2700	28.0
	Fumonisin B_3_	21.4	-	82.5	-	1060	-	-	-	-	-	666	-
	Fumonisin B_4_	-	-	137	19.7	1120	-	25.1	-	11.5	-	891	-
	H. Fumonisin B_1_	5.5	-	-	-	133.5	-	-	-	-	-	70.0	59.6
	Fusarin C	26.1	-	-	-	1050	-	-	-	-	-	363	-
	Bikaverin	21.8	-	17.0	-	957	-	-	-	-	-	170	26.4
	Beauvericin	-	-	-	-	-	-	-	-	-	-	-	-
	Fusaric acid	-	-	-	-	63.4	-	-	-	-	-	-	-
	Fusarinolic acid	-	-	-	-	727	-	-	-	-	-	208	-
	Equisetin	-	-	0.8	3.2	-	-	-	26.8	-	1.2	-	-
B	Alternariol	-	-	-	-	2.9	-	-	-	-	-	8.2	8.5
	Alternariolmethylether	1.9	-	-	-	1.6	0.2	-	-	-	-	3.3	1.8
C	Berkedrimane B	3.6	-	-	-	1.2	-	-	-	-	-	21.6	11.2
	Chrodrimanin	-	-	-	-	65.7	-	-	-	-	-	551	247
	Demethylsulochrin	1.7	-	-	-	3.9	-	-	-	-	-	2.5	8.8
	Penicillide	8.3	-	-	-	26.1	-	-	-	-	-	36.4	28.7
	Pinselin	-	-	-	-	-	-	-	-	-	-	1.8	6.8
	Purpactin A	5.5	1.0	-	-	3.4	1.0	-	-	-	-	22.1	43.3
	Questiomycin A	122	29.3	10.1	-	89.3	31.5	7.5	4.7	479	12.6	126	70.4
	Rugulovasine A	17.7	2.5	2.3	-	7.0	33.7	-	8.0	5.4	2.5	2.6	7.7
	Dehydroaustinol	-	2.7	-	-	5.6	-	-	-	-	-	40.1	16.1
D	Asperglaucide	22.6	21.3	0.1	-	1.6	16.6	0.6	-	-	-	0.3	53.2
	Asperphenamate	53.5	132	0.5	-	0.6	10.0	0.1	-	-	-	0.2	18.4
E	cyclo(l-Pro-l-Tyr)	3.1	2.6	3.8	2.2	-	1.8	1.9	5.4	1.0	2.4	-	4.9
	Emodin	0.3	-	-	-	0.4	-	-	-	-	-	0.4	0.6
	iso-Rhodoptilometrin	0.5	0.2	-	-	1.3	0.1	-	-	-	-	0.8	1.4
	N-B-P	4.0	8.8	-	-	-	-	-	-	-	-	-	3.1
	Tryptophol	27.9	16.8	10.5	15.7	24.9	10.4	-	8.2	-	14.2	26.3	20.4

*H. Fumonisin B*_*1*,_ Hydrolysed fumonisin B_1_; *N-B-P*, *N*-Benzoyl-Phenylalanine

*Indicates conventional (non-GM) maize line

A *Fusarium* sp. metabolites

B *Alternaria* sp. metabolites

C *Penicillium* sp. metabolites

D Unknown sp. metabolites

E Other species

CON indicates the term “conventional”

- Indicates amounts lower than the limit of detection of the equipment

♦ Mycotoxins regulated by EU regulation 1881/2006/EC (sum Fumonisin B_1_ + B_2_ = 4000 μg.kg^−1^)

•• Indicates the respective isogenic GM maize line

## Discussion

The majority of the samples analysed in this study had moisture content (%MC) levels within the safety range for storage without any fungal spoilage. There was no significant difference (*p* = 0.05) in the frequency of isolation of the different fungal genera on the two-culture media (MEA × DG18), although the latter medium selects for more xerotolerant and xerophilic species. *Fusarium* and *Penicillium* spp. were isolated in the highest frequency from all 12 cvs examined, regardless of whether they were GM or non-GM maize. There were also no differences in the dominant species when comparing GM and non-GM cvs. Samples without surface disinfection had significantly higher overall contamination suggesting field and harvesting operations contributed to the inoculum deposited on ripened maize cobs and kernels. *A. flavus* was isolated from a lower percentage of the samples (< 40%) than that with *Fusarium* species. It was interesting to note that strains isolated from non-GM cvs were mostly AFB_1_ producers, whereas the majority of strains from GM cvs were non-toxigenic (Gasperini 2019).

Both phyllosphere field and storage fungi were detected in the directly plated maize samples. Field fungi such as *Cladosporium*, *Alternaria* and especially *Fusarium* species were found in these samples. *Penicillium* spp. were also isolated in high frequency in all the samples. *Aspergillus* sections *Nigri* and *Flavi* were isolated less frequently, with *A. flavus* being detected in eight out of the 12 cvs.

The moisture content and thus *a*_w_ of the maize kernels during the milky ripe stage is very suitable for infection by *Fusarium* species. Subsequently, during the dough stage, the kernels are drier allowing more opportunities for *A. flavus* to colonise, especially where damage has occurred due to the presence of insect pests (Gasperini et al. 2019). Of course, GM cvs which may have insect-related tolerance should in principle have more overall resistance to such mycotoxigenic pathogens (Lacey 1989; Battilani et al. 2011; Gasperini 2019).

No previous studies have examined the secondary metabolite profiles in GM and isogenic non-GM maize. The metabolomic profiles found in these two groups of cvs showed that *Fusarium* metabolites were present in all those examined. It is well-known that *F. verticillioides* can infect maize systemically (Munkvold and Desjardins 1997) and may also survive in an endophytic phase (Alberts et al. 2016) which may contribute to the fumonisins found in these samples. The metabolomic profiles identified was higher in the non-GM maize cvs, including concentrations of fumonisin B_1_ + B_2_ being above the legislative limits.

Fumonisins have often been detected in Brazilian maize (Peluque et al. 2014; Bordin et al. 2015) and even in processed products such as beer (Kawashima and Valente Soares 2006; Piacentini et al. 2017). A high frequency of *Fusarium* spp. (70%) was previously observed in maize hybrids in both asymptomatic and maize with visible kernel rot, resulting in total fumonisins being above the legislation limits (7240 μg.kg^−1^; Lanza et al. 2017). In recent years, there has been a focus on other emerging potentially toxigenic compounds produced by *Fusarium* species such as fusaproliferin, beauvericin, enniatins and moniliformin. Limited data are available on their toxicity (Jestoi 2008), and so far, no firm conclusions have been drawn regarding in vivo toxicity to elaborate a human risk assessment (EFSA 2014). However, some in vitro studies have suggested genotoxic effects of enniatins and beauvericin (Fraeyman et al. 2017). Of course, the impact of mixtures of mycotoxins is now receiving more attention, especially in relation to the potential of synergistic impacts.

In the present study, the occurrence of fusaric acid and fusarin C in the GM and the non-GM maize was low. Previously, fusaric acid and fusarin C contamination of maize was found in at least 50% of the samples analysed by Oliveira et al. (2017) from Brazilian maize, whereas fusarin C has been demonstrated to have mutagenic activity and several immunosuppressive effects comparable with those of AFB_1_ and sterigmatocystin (Cantalejo et al. 1999). Fusaric acid showed low to moderate toxicity, although there are concerns since it might be synergistic with other co-occurring mycotoxins (Bacon et al. 1996).

Despite the higher frequency of isolation of *Penicillium* spp. in the maize samples, regulated mycotoxins were not detected. The major *Penicillia* in cereals (e.g. *P. verrucosum*) prefers cooler temperatures (< 25 °C) and intermediate moisture conditions for ochratoxin A production (Cairns-Fuller et al. 2005) than those occurring in maize cultivation areas of Brazil, and this may explain the absence of this or other related toxins. However, a considerable number of emerging metabolites were present in both GM and non-GM cvs. Questiomycin A and rugulovasine A were the metabolites with the highest occurrence in the samples, being present in 90 and 83%, of samples, respectively. The same metabolites were not detected in the maize from Egypt, but their prevalence was high in feed samples, being 68 and 3%, respectively (Abdallah et al. 2017). Questiomycin A was also detected in many maize samples (94 to 100%) from Serbia in the 2012 to 2015 seasons (Janić Hajnal et al. 2020). In Brazil, *Penicillium* spp. metabolites reported in maize have included rugulovasine A, citrinin, mycophenolic acid, andrastin A, curvularin and penicillic acid, although their prevalence was not high (Oliveira et al. 2017).

It is worthwhile highlighting the differences in mycotoxin profiles between the GM and non-GM maize found in this study. This is in contrast to the frequency of fungal isolation which was similar across the cvs. Some studies have suggested that there are lower mycotoxins in GM maize when compared with non-GM cvs due the reduction of insects that represent an important vector for infection by fungal species of the ripening maize cobs. Associations between insect pests and toxigenic fungi are well*-*known. Mainly Lepidopteran species act as vectors for fungal spores as well as damage the ripening maize kernels, allowing entry of *A. flavus* and other spoilage moulds to infect the cobs (Alma et al. 2005). An effective way to manage Lepidopteran insects and reduce the associated mycotoxin risk has been the use of GM insect*–*resistant genes (Bt) (Munkvold et al. 1999; Wu 2006). Pellegrino et al. (2018) observed mycotoxin contamination in relation to plants expressing resistance to Lepidoptera (GM *Bt*) and this suggested that all stacked *Cry1*Ab hybrids contained significantly less fumonisins and trichothecenes.

Previously, 19 of 23 studies were compared in a review by Ostry et al. (2010) of GM (*Bt*) maize which concluded that this type of maize was less contaminated with *Fusarium* mycotoxins (fumonisins, deoxynivalenol, zearalenone) than the conventional control cvs in each case. However, Naef et al. (2006) suggested that Cry1Ab protein in maize residues has no direct effect on *F. graminearum* and *Trichoderma atroviride*, but some corresponding Bt/non-Bt maize hybrids differed more in composition than the Cry protein content alone, which can affect the saprophytic survival of mycotoxigenic fungi on crop residues.

Barroso et al. (2017) assessed the frequency of *F. verticillioides* and the concentration of fumonisins in GM (*Bt*) and isogenic non-GM hybrids. The GM samples had a lower *F. verticillioides* frequency than non-GM. However, there was no statistical difference between fumonisin contamination when GM *Bt* and non-*GM* samples were compared. The results suggest that other environmental parameters could possibly trigger fumonisin production during plant development in the field.

Junior et al. (2019) correlated the presence of fumonisins in maize with other factors such as the type of hybrid and environment in four different states of Brazil. It showed that high severity of grains infected with *F. verticillioides* does not always result in more fumonisins. Environments with higher temperatures may influence the production of high concentrations of fumonisin in maize (Rosa Junior et al. 2019). Another study of Brazilian maize indicated that a GM hybrid with insect resistance (DKB390 YG) showed greater genetic resistance to the infection by *F. verticillioides* and fumonisin accumulation when compared with the other evaluated hybrids (da Costa et al. 2018). However, within the hybrids used, no comparisons were made between the GM cv and the direct isogenic non-GM line.

Morphological characteristics of the maize kernels in ripening cobs can affect susceptibility to mycotoxin-producing fungi, either directly or indirectly. Hybrids with a thicker kernel pericarp are usually more resistant than those with a thinner pericarp, which can also contribute to resistance. To reduce mycotoxin risk, hybrid selection criteria should include partial resistance to ear rot diseases, appropriate maturity range, husk coverage characteristics and adaptation to local conditions of abiotic stress (Munkvold 2014). da Costa et al. (2018) demonstrated that delaying harvest for minimising drying costs may increase the risk of mycotoxin contamination in maize in the tropics of Brazil. Sampietro et al. (2009) suggested that kernel factors are involved in resistance to fumonisin accumulation in the maize. Resistance was associated with the outer kernel layers and wax content in most of them. Higher wax content would give a broad-based resistance mechanism in maize kernels against mycotoxin production. However, it cannot completely explain the resistance observed.

In summary, this is the first study comparing the fungal contamination and internal infection of GM and non-GM isogenic maize cvs from Brazil containing genetic traits for both insect resistance and/or herbicide tolerance, not only GM *Bt*. The fungal diversity of GM and isogenic non-GM maize grain was found to be very similar (*p* < 0.05). The analysis of the targeted metabolomics profiles of the maize cvs by LC-MS/MS showed higher amounts of *Fusarium* metabolites that paralleled the high isolation frequency of these species from both GM and non-GM maize grain. Overall, the distribution of mycotoxins and related compounds indicated differences between non-GM and GM cvs (*p* < 0.05). GM maize had lower concentrations of different mycotoxins and related secondary metabolites. More detailed studies are now necessary for a better understanding of the potential implications of genetic traits inserted into maize cvs with regard to *A. flavus* colonisation and aflatoxin contamination.
